# Supplementary material for: GPR146 in adipose tissue drives adipose-liver crosstalk and promotes hepatic steatosis in mice
Source: Nat Commun. 2026 Mar 3;17:3389. doi: 10.1038/s41467-026-70136-5 (PMC13065827; doi:10.1038/s41467-026-70136-5)
Supplement: Supplementary file 1 — Supplementary Information [file 41467_2026_70136_MOESM1_ESM.pdf]

SUPPLEMENTARY INFORMATION

Supp_Figure1

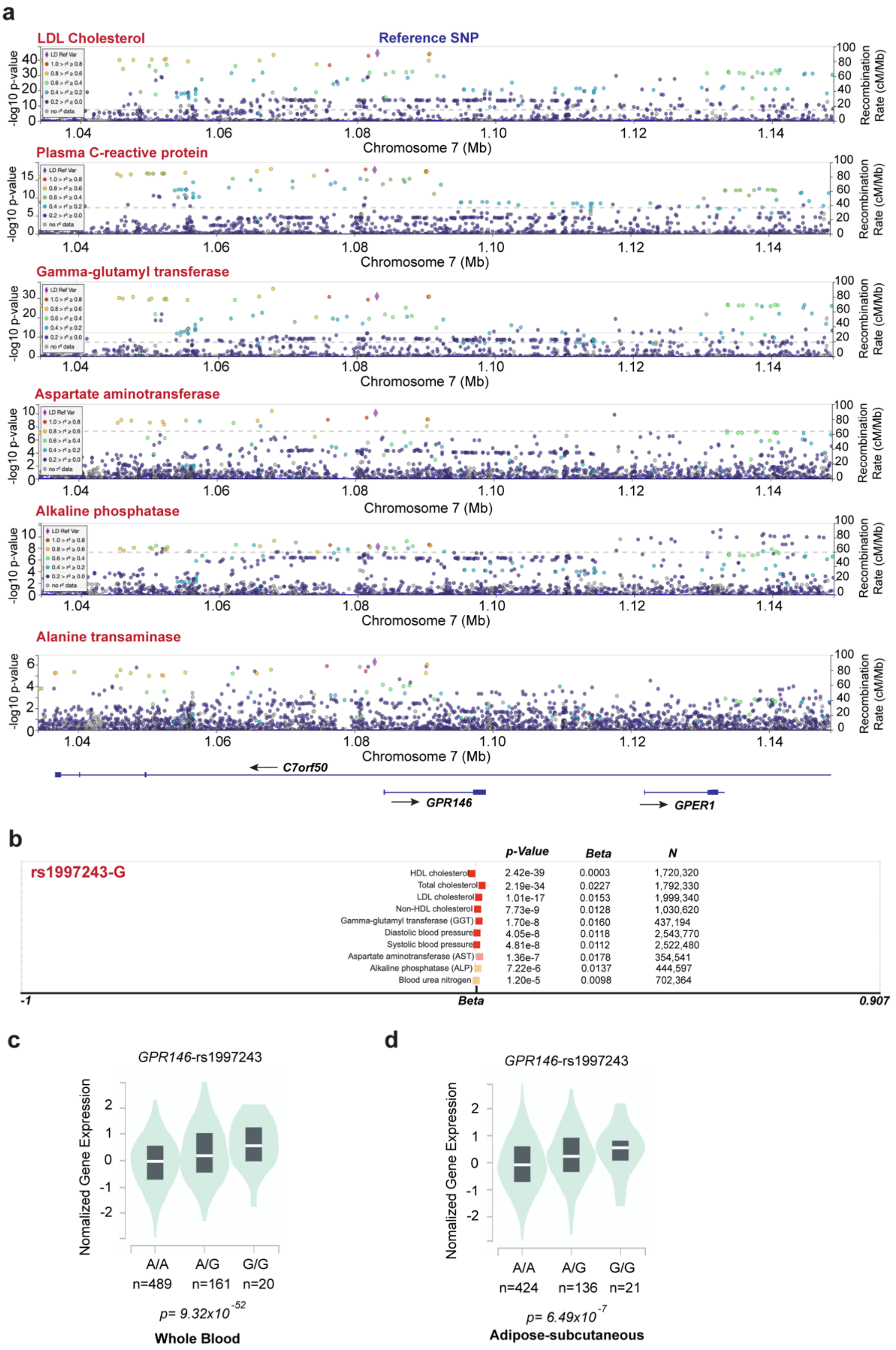

Supplementary Figure 1. Variants in the *GPR146* Gene Are Associated with Plasma Liver Inflammation Enzyme Levels. a, Regional (7p22 locus) association of

GWAS variants with plasma LDL-C, C-reactive protein, gamma-glutamyl transferase (GGT), Aspartate aminotransferase (AST), alkaline phosphatase (ALP), and alanine aminotransferase (ALT) levels in humans. **b,** Lipoproteins, blood pressure, liver inflammation enzymes as a function of the G-allele of rs1997243 in individuals from T2D Portal meta-analysis. The effects are shown as beta-coefficients per G-allele compared with noncarriers. **c,d** eQTL studies using 670 human blood samples (**c**) and 591 human subcutaneous adipose samples (**d**) from GTEx database revealed a highly significant dose-dependent relation between the rs1997243 G-allele and GPR146 expression in both tissues. Source data are provided as a Source Data file.

Supp_Figure2

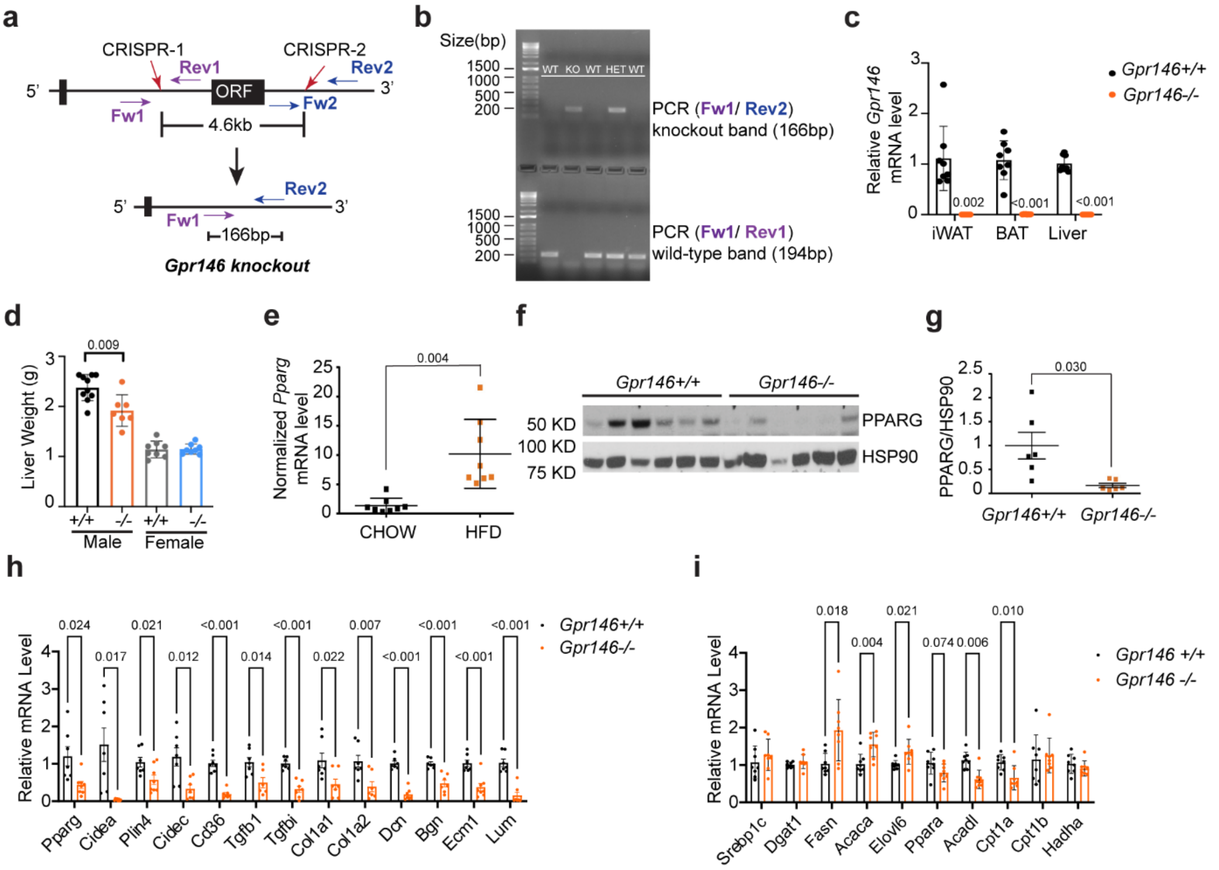

Supplementary Figure 2. GPR146 Deficiency Downregulates PPARγ Signalling Pathway in Mouse Liver, Related to Fig.1. a, Schematic diagram showing the generation of *Gpr146* whole-body knockout mouse model using the CRISPR/Cas9 system, along with the genotyping strategy. **b**, Representative gel images showing PCR products amplified with Fw1/Rev2 and Fw1/Rev1, enabling distinction of wild-type (WT), heterozygous (HET), and homozygous (KO) mice. **c**. qPCR analysis confirming efficient depletion of GPR146 across multiple tissues, including iWAT, BAT, and liver. **d,** Weights of whole liver from male and female *Gpr146* wild-type (+/+) mice and knockout (-/-) littermates fed HFD for 3 months (n=7-10mice per group). **e,** Quantitative polymerase chain reaction (qPCR) expression analysis of *Pparg* mRNA levels in liver of mice fed chow or HFD for 3 months (n=8 mice per group). **f, g,** Western blot (**f**) and relative quantification (**g**) of PPARG in liver of 16 h fasted male *Gpr146*$^{+/+}$ mice and *Gpr146*$^{-/-}$ littermates fed HFD for 3 months (n=6 mice per group). **h,** qPCR expression analysis of adipogenesis and extracellular matrix genes in liver of 16 h fasted male *Gpr146*$^{+/+}$ and *Gpr146*$^{-/-}$ littermates fed HFD for 3 months (n=8 mice per group). **i**, qPCR expression analysis of fatty acid biosynthesis or b-oxidation genes in

liver of 16 h fasted male *Gpr146*$^{+/+}$ and *Gpr146*$^{-/-}$ littermates fed HFD for 3 months (n=8 mice per group). Bars in c, d and e indicate mean ± s.d., bars in g-i indicate mean ± s.e.m.. Statistical analyses were performed using two-sided unpaired t-tests, with P values (c–e, g–i) indicated. Source data are provided as a Source Data file.

Supp_Figure3

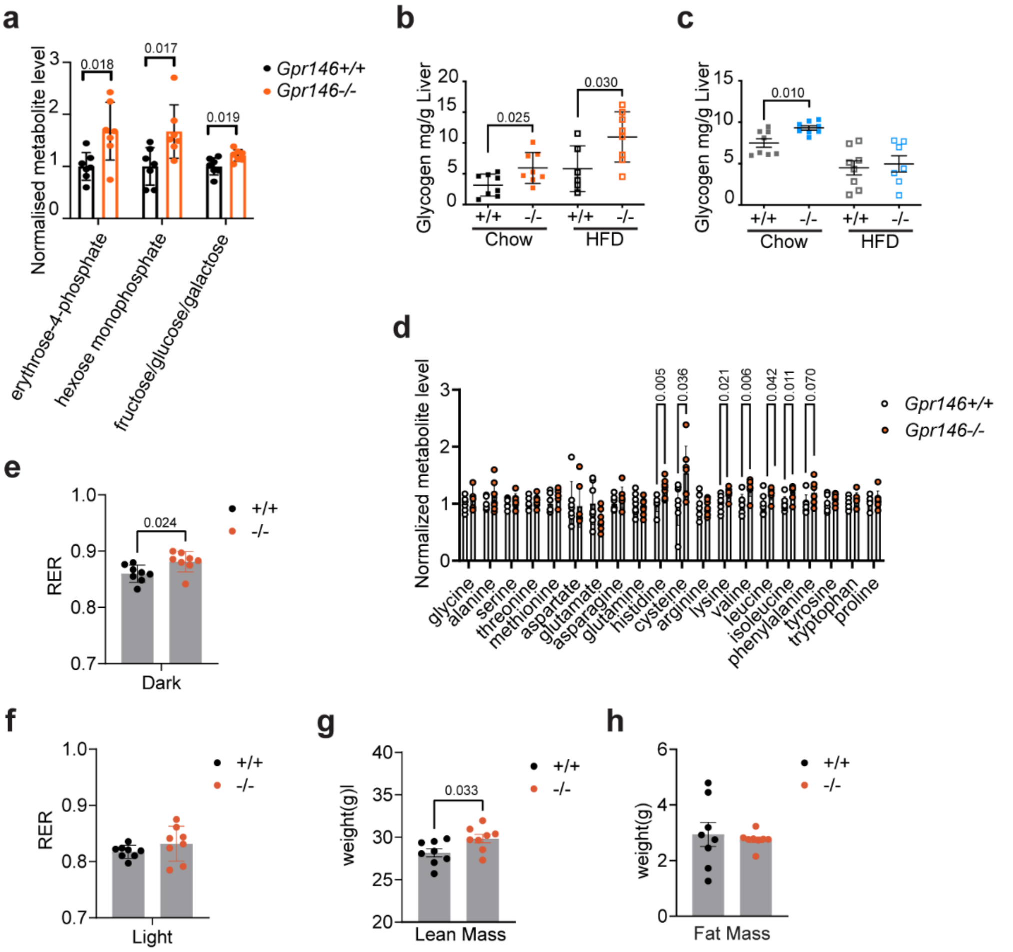

Supplementary Figure 3. GPR146 deficiency reprograms hepatic glucose and amino acid metabolism. a, Hepatic erythrose-4-phosphate, hexose monophosphate, and hexose of 16 h fasted male *Gpr146* wild-type (+/+) mice and knockout (-/-) littermates fed an HFD (n=7 mice per group). **b,c,** Hepatic glycogen content of 16 h fasted male (**b**) and female (**c**) *Gpr146* wild-type (+/+) mice and knockout (-/-) littermates fed chow or HFD as indicated (n= 8-13 mice per group). **d**, Relative abundance of hepatic amino acids in 16 h fasted male mice fed an HFD (n=7 mice per group). **e.f**, Respiratory exchange ratio (RER) measured during the **dark** (**e**) and **light** (**f**) phases by indirect calorimetry in chow-fed *Gpr146* $^{+/+}$ and *Gpr146* $^{-/-}$ male mice. **g,h**, Magnetic resonance imaging (MRI) analysis of lean mass (**g**) and fat mass (**h**) of *Gpr146*$^{+/+}$ and *Gpr146*$^{-/-}$ littermates on chow diet. Bars in a-d indicate mean ± s.d., bars in e-h indicate mean ± s.e.m.. Statistical analyses were performed using two-sided unpaired t-tests, with P values indicated. Source data are provided as a Source Data file.

Supp_Figure4

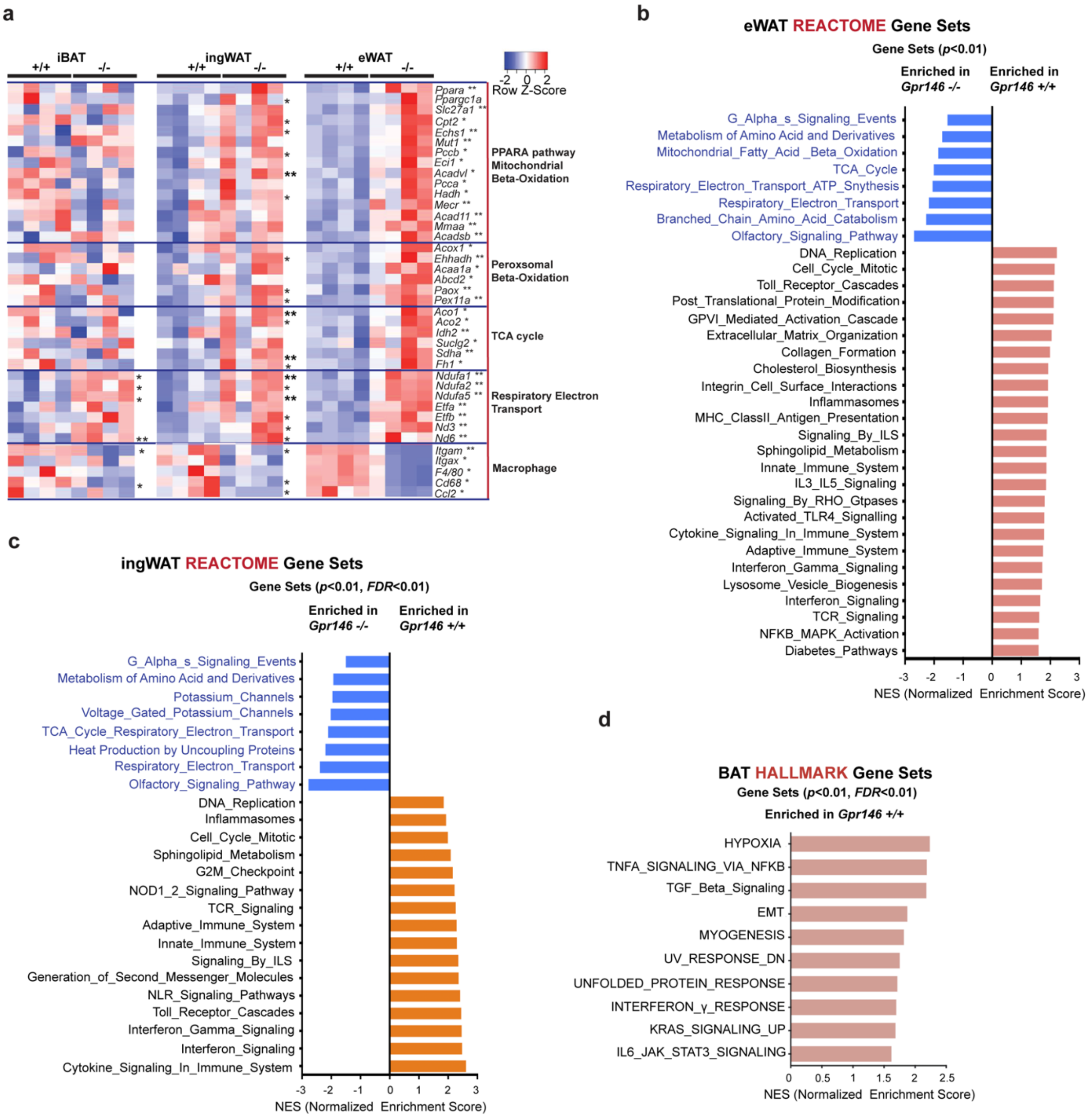

Supplementary Figure 4. Transcriptomic remodeling and pathway alterations in adipose tissues associated with GPR146 deficiency, Related to Fig.2. a. Heatmap of Beta-oxidation, TCA cycle, and electron transport chain (HALLMARK Pathway Database)-related genes in ingWAT, eWAT, and iBAT of male *Gpr146*$^{+/+}$ and *Gpr146*$^{-/-}$ littermates fed HFD for 3 months (n=4 per group, by two-sided unpaired t-test). **b-d,** Top ranking REACTOME or HALLMARK pathway gene sets discovered from gene set enrichment analysis (GSEA) of genes that are differentially expressed in eWAT (**b**), iWAT (**c**), and BAT (**d**) of 16 h fasted male *Gpr146*$^{+/+}$ and *Gpr146*$^{-/-}$ littermates fed

HFD for 3 months (n=4 mice per group), with significance assessed by permutation-based testing and false discovery rate (FDR) correction. *, $p < 0.05$, **, $p < 0.01$.

Supp_Figure5

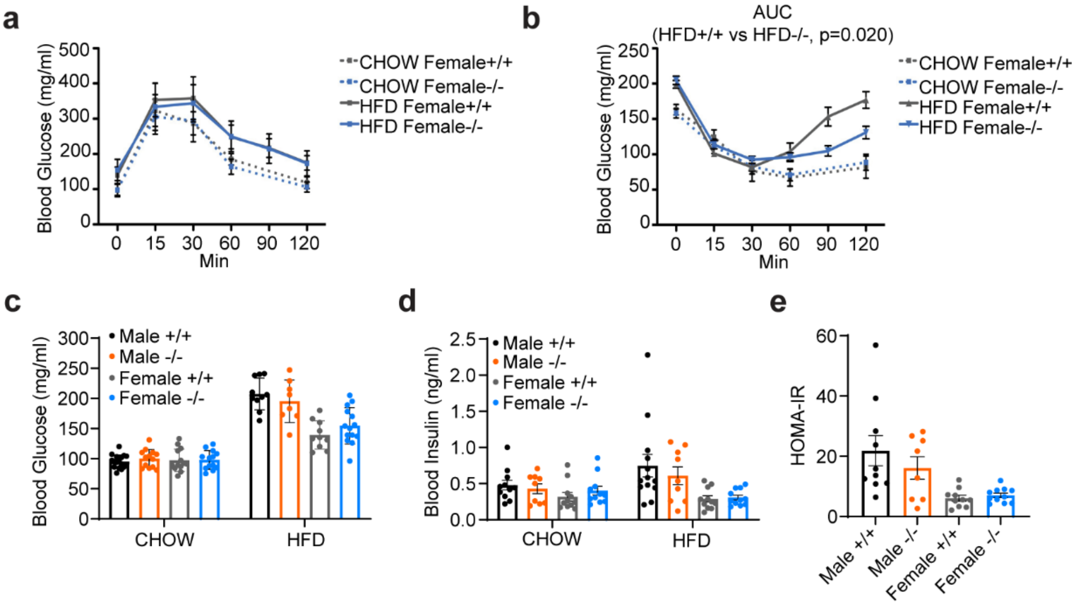

Supplementary Figure 5. GPR146 deficiency alters systemic glucose metabolism, Related to Fig.2. a,b, Plasma glucose during glucose tolerance test (**a**) and insulin tolerance test (**b**) of female *Gpr146*$^{+/+}$ and *Gpr146*$^{-/-}$ littermates fed chow or HFD for 2 months (n=10-15 mice per group). **c,d,** Plasma glucose (**c**) and insulin (**d**) levels of 16 h fasted mice fed chow or HFD for 2 months (n=7-10 mice per group). **e.** HOMA-IR calculated from fasting glucose and insulin levels in mice fed HFD for 1 month (n = 8–11 mice per group). Bars in a, b and e indicate mean± s.e.m.; bars in c and d indicate mean ± s.d.. Statistical analyses were performed using two-sided unpaired t-tests(c-e). For panel a, b, AUC was calculated and compared between HFD-fed female *Gpr146* $^{+/+}$ and *Gpr146* $^{-/-}$ mice using two-sided unpaired t-test with P value indicated. Source data are provided as a Source Data file.

Supp_Figure6

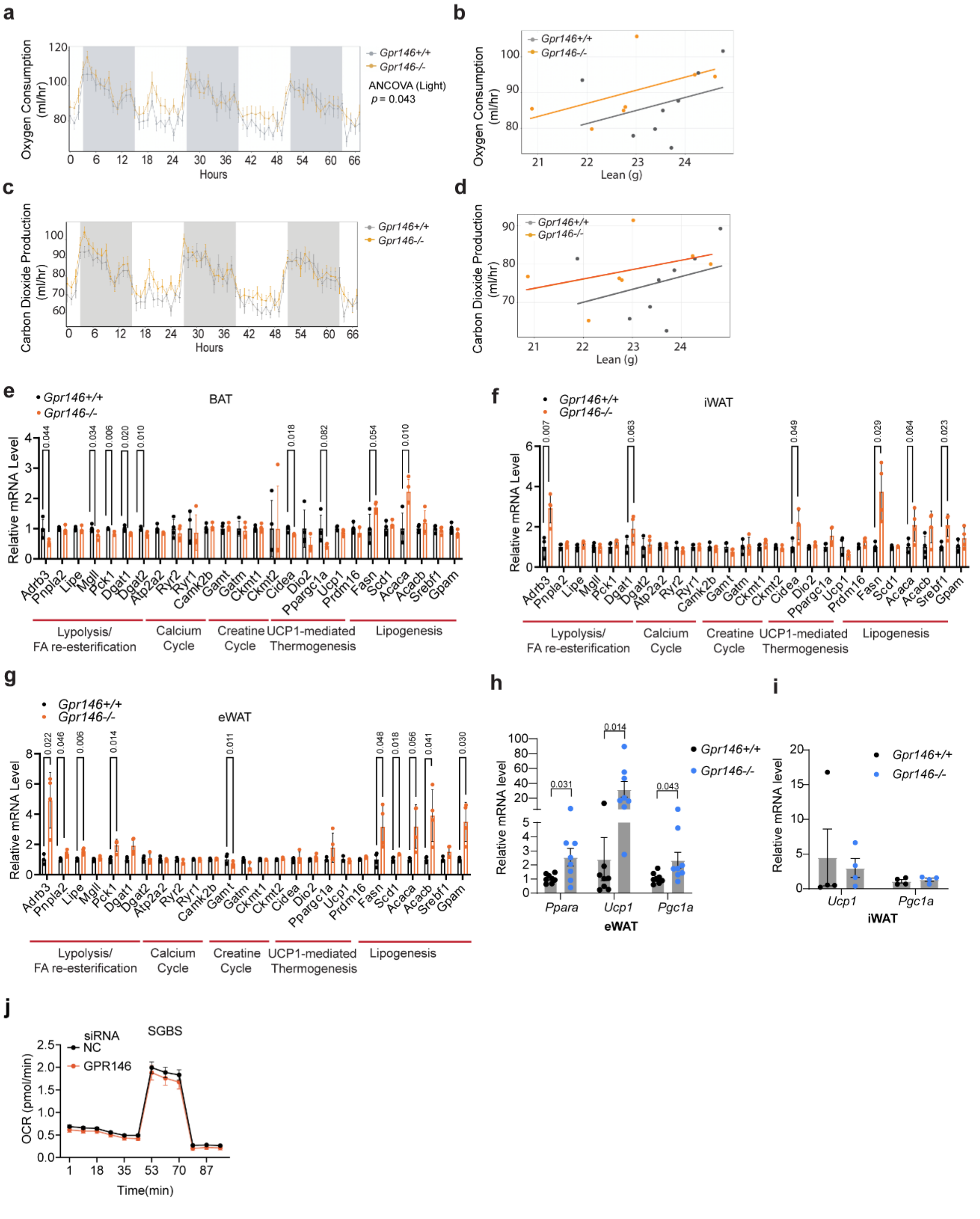

Supplementary Figure 6, Metabolic phenotyping and thermogenic pathway regulation in adipose tissue of GPR146-deficient and control mice, Related to Fig3. a-d, Oxygen consumption (**a**), regression plots for analysis of oxygen consumption versus lean mass (**b**), carbon oxygen consumption (**c**), and regression plots for analysis of carbon oxygen consumption versus lean mass (**d**) for *Gpr146*$^{+/+}$ and *Gpr146*$^{-/-}$ littermates (n = 8 mice per group). **e-g,** Relative mRNA expression levels

of selected genes associated with three major thermogenic pathways in BAT (**e**), iWAT (**f**), eWAT (**g**) of male *Gpr146*$^{+/+}$ and *Gpr146*$^{-/-}$ littermates fed HFD for 3 months (n=4 per group)**. h,i,** qPCR expression analysis of *Ucp1, Pgc1a* and *Ppara* in eWAT (**h**) and iWAT (**i**) from female *Gpr146*$^{+/+}$ and *Gpr146*$^{-/-}$ littermates fed HFD (n=4-8 mice per group). **j**, Oxygen consumption rate (OCR) measured by Seahorse XF mitochondrial stress test in SGBS adipocytes transfected with control (NC) or GPR146 siRNA. Bars in a-d, h and i indicate mean± s.d.; bars in e,f and g indicate mean ± s.e.m.. Statistical significance was determined by two-way ANCOVA (a-d), two-sided unpaired t-tests (e–j), with adjusted P values or P values indicated. Source data are provided as a Source Data file.

Supp_Figure7

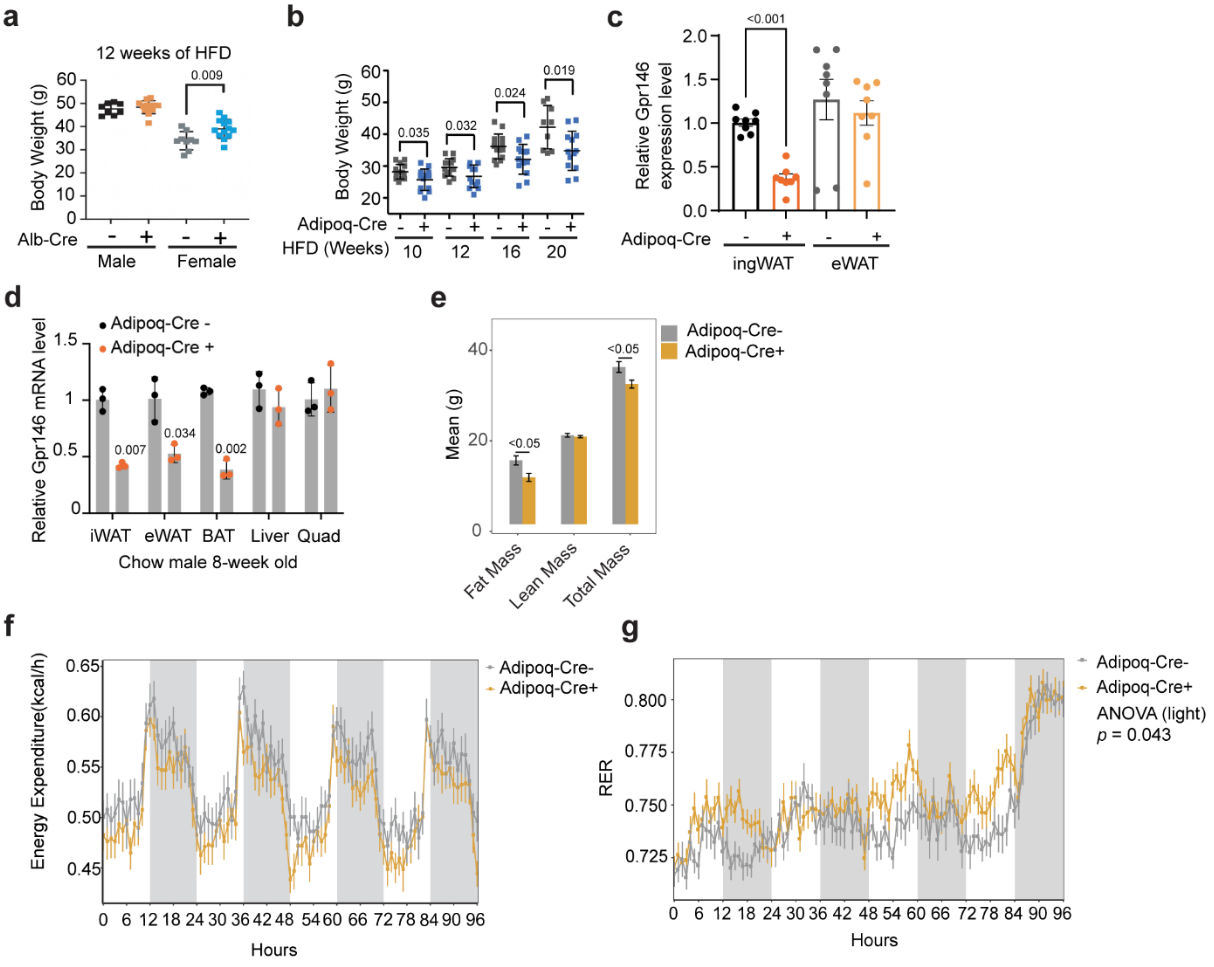

Supplementary Figure 7, Adipose GPR146 mediates protection against diet-induced obesity and liver steatosis, Related to Fig.4. a, Body weight of male or female liver-specific knockout mice (Alb-Cre^{+}) and control littermates (Alb-Cre^{-}) fed HFD for 3 months (n = 8-15 mice per group). **b,** Body weight of female adipose-specific knockout mice (Adipoq-Cre^{+}) and control littermates (Adipoq-Cre^{-}) fed HFD as indicated (n = 9-14 mice per group). **c,d**, Relative expression of *Gpr146* mRNA in ingWAT and eWAT from male mice at 8-week-old (**d**) and after fed HFD for 4 months (**c**) (n = 8 mice per group). **e,** MRI analysis of fat mass and lean mass from female adipose-specific knockout mice (Adipoq-Cre^{+}) and control littermates (Adipoq-Cre^{-}) fed HFD for 5 months. **f,** Energy Expenditure measured by indirect calorimetry in Adipoq-Cre^{+} and control mice (Adipoq-Cre^{-}) fed HFD for 4 months. **g**, Hourly respiratory exchange ratio (RER) measured in the same cohort of male Adipose specific knockout and the control littermates. Bars in all panels indicate mean± s.d..

Statistical significance was determined by two-sided unpaired t-tests (a–d) or two-way ANCOVA (e–g), with P values or adjusted P values indicated. Source data are provided as a Source Data file.

Supp_ Figure8

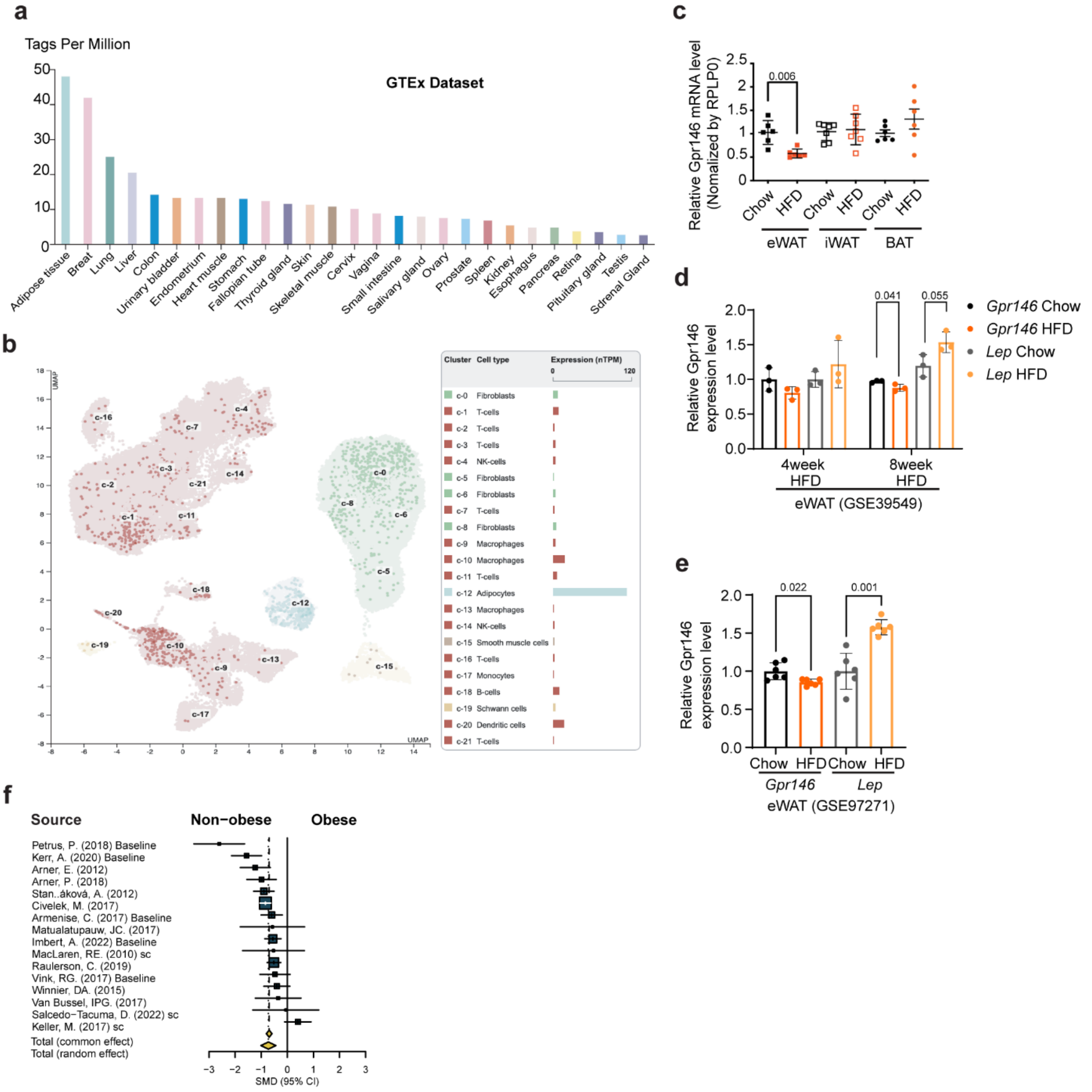

Supplementary Figure 8, GPR146 expression in adipose tissue is modulated by nutritional and physiological context in mice and humans, Related to Fig.4. a, Profiling of GPR146 expression in a variety of human tissues (The Human Protein ATLAS). **b**, Single cell profiling of *GPR146* expression in human adipose tissue (The Human Protein ATLAS). **c**, *Gpr146* mRNA expression in eWAT, iWAT, and BAT from WT C57/BL6 mice fed a chow or high-fat diet (HFD) for 3 months (n = 6–10 per group). **d, e,** Analysis of mouse eWAT transcriptomic data from two publicly available datasets, GSE39549 (**d**) and GSE97271 (**e**), comparing Gpr146 expression before and after HFD feeding, and its relationship with *Lep* (leptin) expression**. f,** Forest plots

summarizing GPR146 expression across multiple human transcriptomic cohorts (data adapted from the Adipose Tissue Knowledge Portal), comparing individuals with and without obesity. Source studies included in the meta-analysis are listed alongside each plot. Standardized mean differences (SMD) with 95% confidence intervals were calculated for each cohort and combined using a random-effects meta-analysis. Bars in d, e indicate mean ± s.e.m.. Statistical significance was determined by two-sided unpaired t-tests (c–e), with P values indicated. Source data are provided as a Source Data file.

Supp_Figure9

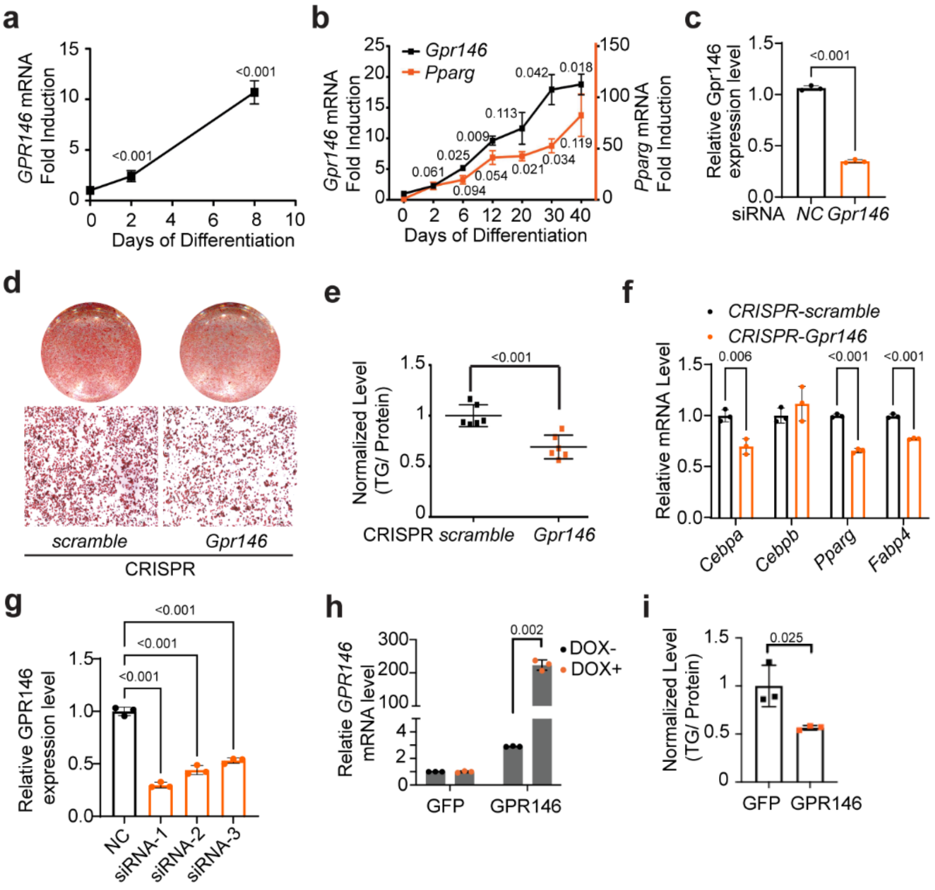

Supplementary Figure 9, GPR146 promotes differentiation of both mouse and human preadipocytes, Related to Fig.6. a, Relative expression of *GPR146* messenger RNA during adipocyte differentiation of SGBS pre-adipocytes (n=3 replicates per experiment, representative of 3 independent experiments). **b**, Relative expression of *Gpr146* and *Pparg* messenger RNA during adipocyte differentiation of SVF-derived pre-adipocytes (n=3 replicates per experiment, representative of 3 independent experiments). **c,** Relative expression of *Gpr146* mRNA in mouse SVF upon siRNA-induced knockdown of *GPR146* (n=3 replicates per experiment, representative of 3 independent experiments). **d,e,** Oil Red O-staining (**d**) and TG content (**e**) of mouse SVF-differentiated adipocytes upon CRISPR/Cas9-based knockout of *Gpr146* (n=3 replicates per experiment, representative of 3 independent experiments). **f,** Quantitative qPCR expression analysis of adipogenesis genes at day 4 of adipocyte differentiation (n=3 replicates per experiment, representative of 3 independent experiments). **g,h**, Relative *GPR146* mRNA expression in human SGBS cells following siRNA-mediated knockdown (**g**) or doxycycline-induced overexpression

of *GPR146* (**h**) (n = 3 replicates per condition, representative of 3 independent experiments). **i**, Triglyceride content in SGBS-differentiated adipocytes overexpressing *GPR146* or GFP (control). Bars in a-e, g, h, and i indicate mean± s.d., bars in f indicate mean ± s.e.m.. Statistical significance was determined by two-sided unpaired t-tests (a,c and e–i) or two-way ANOVA (b), with P values or adjusted P values indicated. Source data are provided as a Source Data file.

Supp_Figure10

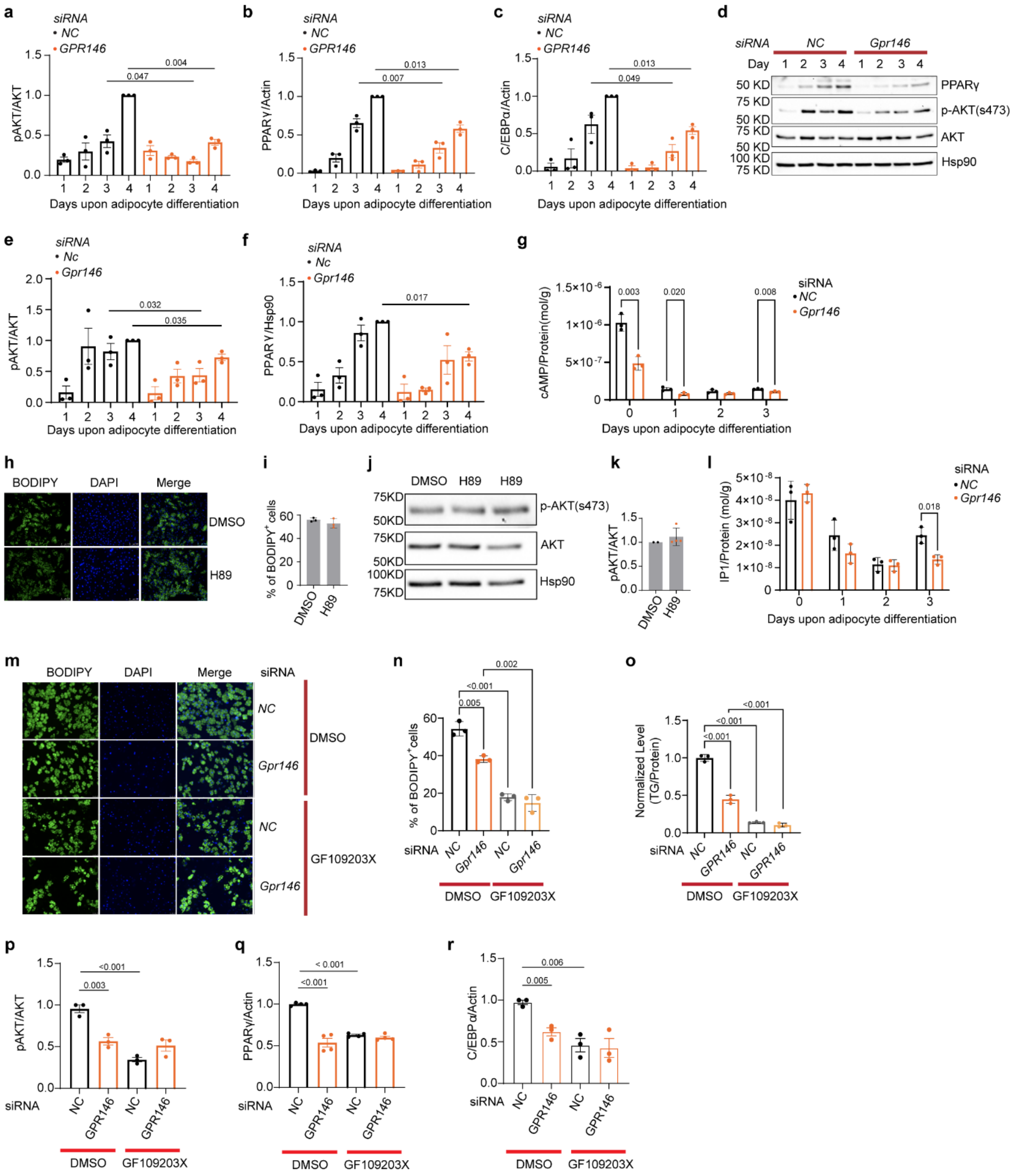

Supplementary Figure 10, GPR146 Regulates Adipogenesis through Gαq-mediated Activation of PKC Signaling Pathway in both human and mouse preadipocytes, Related to Fig.7.a-c, Relative quantification of western blots (Fig. 7c) for p-AKT (s473), AKT, PPARγ, C/EBPα and Actin in *GPR146* knockdown or control SGBS pre-adipocytes upon differentiation for the time indicated. **d-f,** Representative western blots (**d**) and relative quantification for p-AKT (s473), AKT (**e**), and PPARγ (**f**)

in *Gpr146* knockdown or control SVF pre-adipocytes upon differentiation for the time indicated. **g,** Cellular cAMP level in *Gpr146* knockdown or control SVF pre-adipocytes upon differentiation for the time indicated (n=3 replicates per experiment, representative of 3 independent experiments). **h,i,** BODIPY-staining (**h**) and percentage of BODIPY+ cells (**i**) of mouse SVF-differentiated adipocytes in the presence or absence of H89 (n=3 replicates per experiment, representative of 3 independent experiments). **j,k,** Representative western blots (**j**) and relative quantification(**k**) of p-AKT (s473) and ATK in SVF at day 4 of adipocyte differentiation in the presence or absence of H89 (representative of 2 independent experiments). **l**, Cellular IP1 levels in *Gpr146* knockdown or control SVF pre-adipocytes upon differentiation for the time indicated (n=3 replicates per experiment, representative of 3 independent experiments). **m-o** BODIPY-staining (**m**), percentage of BODIPY+ cells (**n**), and triglyceride (TG) content (**o**) of mouse SVF-differentiated adipocytes upon siRNA-induced knockdown of *Gpr146* in the presence or absence of GF109203X (n=3 replicates per experiment, representative of 3 independent experiments). **p-r**, Relative quantification of western blot (**Fig. 7h**) for p-AKT(s473), AKT (**p**), PPARγ (**q**), C/EBPα (**r**), and Actin (**q, r**) in GPR146 knockdown or control SGBS pre-adipocytes upon differentiation for 4 days in the presence or absence of GF109203X. Bars in a, b, c, e, f, k, p, q and r indicate mean± s.e.m., bars in g, i, l, n and o indicate mean± s.d.. Statistical significance was determined by two-sided paired tests (a-c, e and f) unpaired t-tests (g, i, k and l) or two-way ANOVA (n–r), with P values or adjusted P values indicated. Source data are provided as a Source Data file.

Supp_Figure11

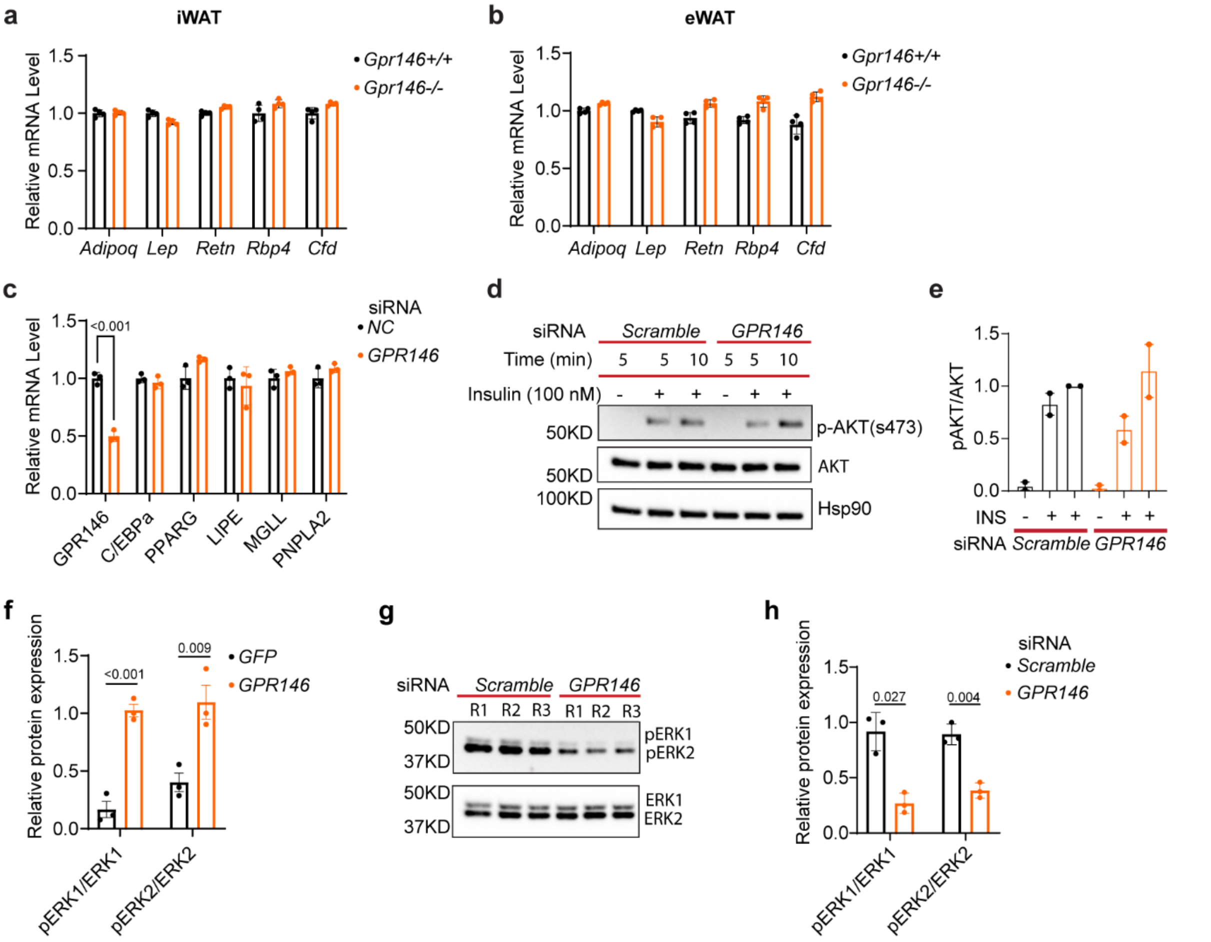

Supplementary Figure 11, GPR146 regulates mature adipocyte lipolysis through ERK signaling, Related to Fig. 8. a,b, Relative mRNA expression of genes encoding major adipokines in iWAT(**a**) and eWAT(**b**) from male *Gpr146*$^{+/+}$ and *Gpr146*$^{-/-}$ littermates fed HFD for 3 months (n=4 mice per group)**. c,** qPCR expression analysis of key transcriptional regulators of adipogenesis and lipases involved in lipolysis in mature adipocyte with *GPR146* knockdown by siRNA (n=3 mice per group). **d**,**e**, Representative western blots (**d**) and relative quantification (**e**) of p-AKT (s473) and AKT in mature adipocytes derived from SGBS cells following *GPR146* knockdown and Insulin stimulation (100nM) at indicated time points. **f**, Relative quantification of western blot (**Fig. 8d**) for phosphorylated ERK (p-ERK) and total ERK in SGBS cells with doxycycline-induced GFP or GPR146 expression. **g**,**h**, Representative western blots (**g**) and relative quantification (**h**) of p-ERK and ERK in *GPR146* knockdown SGBS-derived mature adipocytes. Bars in a, b and c indicate mean± s.d.. bars in e, f and h indicate mean±s.e.m.. Statistical significance was determined by two-sided

unpaired t-tests (a–c), paired t-tests (f and h), with P values indicated. Source data are provided as a Source Data file.

Supplementary Table 1

REAGENT or RESOURCE	SOURCE	IDENTIFIER
**Antibodies**		
Mouse monoclonal anti-HSP90	Santa Cruz Biotechnology	Cat# sc-13119; RRID: AB_ 675659
Rabbit polyclonal anti- Akt	Cell Signaling Technology	Cat#9272; RRID:AB_ AB_329827
Rabbit monoclonal anti-phospho-Akt(Ser473)(D9E)	Cell Signaling Technology	Cat#9271; RRID:AB_2315049
Rabbit monoclonal anti-PPARγ (C26H12)	Cell Signaling Technology	Cat# 2435
Rabbit polyclonal anti-C/EBPα [EP708Y]	Abcam	Cat#ab40761; RRID:AB_1140761
Mouse monoclonal anti-C/EBPα(D5)	Santa Cruz Biotechnology	Cat#sc-365318; RRID:AB_10846948
Mouse monoclonal anti-β-actin	Sigma-Aldrich	Cat# A5316; RRID: AB_ 476743
Rabbit monoclonal anti-p44/42 MAPK (Erk1/2)	Cell Signaling Technology	Cat# 4695; RRID:AB_390779
Rabbit monoclonal anti-phospho-p44/42 MAPK (Erk1/2) (Thr202/Tyr204)	Cell Signaling Technology	Cat# 4370; RRID: AB_ 2315112
**Chemicals**		
PKC inhibitor (GF109203X)	Sigma-Aldrich	Cat#G2911
Dexamethasone	Sigma-Aldrich	Cat#D1756
Insulin	Sigma-Aldrich	Cat# I9278
3-Isobutyl-1-methylxanthine (IBMX)	Sigma-Aldrich	Cat#I5879
Rosiglitazone	Sigma-Aldrich	Cat#R2408
Panthotenat	Sigma-Aldrich	Cat#P5155
Cortisol	Sigma-Aldrich	Cat#H0888
Triiodothyronine (T3)	Sigma-Aldrich	Cat# T6397
BODIPY® 493/503	Thermo Fisher Scientific	Cat# D3922
Hoechst 33342	Thermo Fisher Scientific	Cat# 62249
Oil Red O	Sigma-Aldrich	Cat# O0625
Eosin Y solution	Sigma-Aldrich	Cat# 318906
Hematoxylin Solution, Mayer′s	Sigma-Aldrich	Cat# MHS32
PD0325901	Selleckchem	Cat#S1036
CL316,243	MCE	Cat#HY-116771A
Collagenase A	Sigma-Aldrich	Cat#H3023
PD98059	Selleckchem	Cat#S1036
Foskolin	Sigma-Aldrich	Cat#F6886
**Critical Commercial Assays**		
Infinity Triglycerides Reagent	Thermo Fisher Scientific	Cat# TR22421
Infinity Triglycerides Reagent	Thermo Fisher Scientific	Cat# TR13421
cAMP-Gs dymanic kit	PerkinElmer	Cat# 62AM4PEB
IP-One Gq Kit	PerkinElmer	Cat# PKE_62IPAPEB
ALT kit	Sigma-Aldrich	Cat# MAK052
BrdU Cell Proliferation Assay Kit	Cell Signaling Technology	Cat# 6813
Free Glycerol Reagent	Sigma-Aldrich	Cat# F6428
Mouse insulin ELISA kit	Abcam	Cat# ab277390
**Deposited Data**		
Microarray raw data	This paper	GEO(GSE316218)
**Experimental Models: Cell Lines**		

Human: Human Simpson–Golabi–Behmel syndrome (SGBS) cell line	University of Ulm, Germany	
**Experimental Models: Organisms/Strains**		
Mouse: Gpr146$^{-/-}$	This paper	N/A
Mouse: B6.Cg-Speer6-$^{ps1Tg(Alb-cre)21Mgn}$/J	The Jackson Laboratory	RRID: IMSR_JAX:003574
Mouse: B6.FVB-Tg(Adipoq-cre)1Evdr/J	The Jackson Laboratory	RRID: IMSR_JAX:010803
Mouse: Gpr146-LKO	This paper	N/A
Mouse: Gpr146-FKO	This paper	N/A
Mouse: B6J.129(Cg)-Gt(ROSA)26Sor$^{tm1.1(CAG-cas9*,-EGFP)Fezh}$/J	The Jackson Laboratory	RRID: IMSR_JAX:026179
Mouse : C57BL6/J	The Jackson Laboratory	RRID: IMSR_JAX:000664
**Oligonucleotides (5' to 3' orientation)**		
Mouse *Gpr146* sgRNA1 sequence (for generating whole-body knockout mouse model): GCTACCTTTTGGCACGGGTT	This paper	N/A
Mouse *Gpr146* sgRNA2 sequence (for generating whole-body knockout mouse model): GTGGCTAAGGGATTATATGG	This paper	N/A
Mouse *Gpr146* sgRNA1 sequence (for knockout of *Gpr146* in adipose-derived SVF cells): GCCCACGCTGTGCTGTTGAG	This paper	N/A
Mouse *Gpr146* sgRNA2 sequence (for knockout of *Gpr146* in adipose-derived SVF cells): CCCCCAGGTAGAGCAGCGAG	This paper	N/A
Mouse *Gpr146* sgRNA3 sequence (for knockout of *Gpr146* in adipose-derived SVF cells): GTACTTCGTGAACATGGCCG	This paper	N/A
Mouse *Gpr146* siRNA	Thermo Fisher Scientific	s206895
Human *GPR146* siRNA	Thermo Fisher Scientific	s41795
Mouse *Gpr146* AAV8-shRNA targeting sequence: GCATTATC TGGGCATCCTACA	This paper	N/A
**Software and Algorithms**		
ImageJ	NIH	https://imagej.nih.gov/ij/
GraphPad Prism9	GraphPad Software	https://www.graphpad.com/
Adiposoft	[46]	
KSEA	[37]	

Supplementary Table 2: qPCR primers

Mouse

Gene	Forward	Reverse
Gpr146	ACGCACCTACATGGCCAGTGTGTAC	CGATTCTAGAAGACACGTGACTGCAGATGT
Rplp	GAAGACAGGGCGACCTGGAAGTCCAACTAC	GATCTGCTGCATCTGCTTGGAGCCCAC
Pparg	TTCACAAGAGCTGACCCAATGGTTGCTGATTAC	ATGAGGCCTGTTGTAGAGCTGGGTCTTTTCAG
Cidea	AGTTTCAAACCATGACCGAAGTAGCCGGCG	ACCAGCGTAACCAGGCCAGTTGTGATGAC
Plin4	GATGAGTTGAAAGGGCTGGGTGATATCTTTC	GACGGATGTAGTAGCTTCCCCGGTCAGC
Cd36	GAGCCTTCACTGTCTGTTGGAACAGAGGATG	ATAAGAGAGTTGAGCACAACTTGAACAAATG
Col1a1	ATGTTCAGCTTTGTGGACCTCCGGCTCC	GTTTCCACGTCTCACCATTGGGGAC
Col1a2	GAGGCAGAGATGGTGTTGATGGTCCCATG	ATTGGTCCAGGGCCAGATGAAACTCCTTTG
Il6	CCACTTCACAAGTCGGAGGCTTAATTACAC	CAGATTGTTTTCTGCAAGTGCATCATCGTTG
Tnfa	TGCCTATGTCTCAGCCTCTTCTCATTCCTGC	TGAGTGTGAGGGTCTGGGCCATAGAACTG
Il1b	GCCCATCCTCTGTGACTCAT	AGGCCACAGGTATTTTGTCG
Cebpa	CAAAGCCAAGAAGTCGGTGGACAAGAACAG	CCTTCTGTTGCGTCTCCACGTTGCGTTG
Cebpb	AAGCTGAGCGACGAGTACAAGATGCGG	TCGTTCTCCGCCGTCAGCTCCAGC
Fasn	TGGTGTGGACATGGTCACAGATGATGACAGG	TGGACCCCAAAAAAGGAGGCGTCGAAC
Fabp4	TAAAAACACCGAGATTTCCTTCAAACTGGG	ATTGTGGTCGACTTTCCATCCCACTTCTGC
F4/80	TTGGGATCTGCCCTAAGTATTCCAACTGC	AGGGCAAACGTCTCGAGTCACACATTC
Mcp1	ATGATCCCAATGAGTAGGCTGGAGAGCTACAAG	ATTCCTTCTTGGGGTCAGCACAGACCTC
Lpl	GAGTTTGGCTCCAGAGTTTGACCGCCTTCC	AGCTGTGTCTTCAGGGGTCCTTAGGGC
Ppara	TGCAGACTACCAGTACTTAGGAAGCTGTCC	GACTGAGGAAGGGCTGGAAGCTGGAGAGAG
Adipoq	GCAGAGATGGCACTCCTGGAGAGAAGGG	CCTTCAGCTCCTGTCATTCCAACATCTCC
Tgfb1	TACCAGCTGGTGCTGCAGCATAGC	CTTCCTCTGGTACCACTGCTTGCAGTTG
Dcn	GATGCCAGTGTCATCTTCGAGTGGTGCAGT	ATGCAAGTCCTTCAGGTTCTTGAAGGCCC
Bgn	GACTCTGTCACACCTACCTTCAGTGCCATG	TCTGCAGGTCTAGCAGTGTGGTGTCAGGTG
Ecm1	GACCGTATCCAGAGCAGCCTTGATCTTGGC	CTTCATGGAGGTGCTGGAAGAGGCGCTC
Lum	GAGTATACCAACAGTTAATGAAAATCTTG	GCTTGATCTTGGAGTAAGACAGTGGTCCCA
Srebp1c	TGACAGGTGAAATCGGCGCGGAAGCTG	GTCTTGGTTGTTGATGAGCTGGAGCAT
Dgat1	AATCATCTGCTTCCCAGCAGCTGTGGCC	CACCACAGGTTGACATCCCGGTAGGAATAAAG
Acaca	CAGATACACTTTCTGATTTGGGGATCTCTG	GTGAGTCTATTTTCTTTCTGTCTCGACCTTG

Elovl6	GAATTCGAAAAGCAGTTCAACGAGAACGAAG	GATGCCGACCACCAAAGATAAAGGCAGC
Acadl	CGGGAGAGTGTAAGGAAGTTTTTCCAAGAAG	ATGTTGATGCCAAGCAAGCCCTGCTTGC
Cpt1a	CAAACAGATCTGCCTGTCAGGGCTGCACT	GTATGCATGGATGATATCACACCCACCACC
Cpt1b	CGGGAGGCTCTGAGACACATCTACCTG	GTAGTTGGAACCAACTGTTGCCATGACAAC
Hadha	TGCATTTGCCGCAGCTTTAC	GTTGGCCCAGATTTCGTTCA
Ucp1	GGAGTTTCAGCTTGCCTGGCAGATATCATC	GTGATGGTCCCTAGGACACCTTTATACCTAATG
Pgc1a	CAGCTCAGCTACAATGAATGCAGCGGTC	AATGCTCTTCGCTTTATTGCTCCATGAATTC

Human

Gene	Forward	Reverse
GPR146	ACACAGCAAGGCCAGCATGACCATG	TGGACTTCGCCGCCCACACTCCAC
RPLP	CGACCTGGAAGTCCAACTACTTCCTTAAG	CATGCGGATCTGCTGCATCTGCTTGGAG
CEBPA	GGTGCTGGAGCTGACCAGTGACAATGAC	AGTTGCCCATGGCCTTGACCAAGGAGC
CEBPB	GACAAGCACAGCGACGAGTACAAGATCC	TTCTCGGCCGTGAGCTCCAGGACCTTG
PPARG	ACAAGAACAGATCCAGTGGTTGCAGATTAC	CATGAGGCTTATTGTAGAGCTGAGTCTTCTC
FASN	CAAGCAGGCACACACGATGGACCCTCAG	CGCCAGTGTGTGTTCCTCGGAGTGAATC
FABP4	GAAGTAGGAGTGGGCTTTGC	TTCCTGGCCCAGTATGAAGG
LIPE	CCGACTGAATCTACGTCCCA	TAGGGCTGATCGCTGTATGG
MGLL	AAAGACTACCCTGGGCTTCC	CAGCACAAGGTTGAGCACTT
PNPLA2	AAGGTCCTGCCTGCTGATAG	GATGAAACCGCTGCAGACAT
