# Supplementary material for: GPR146 in adipose tissue drives adipose-liver crosstalk and promotes hepatic steatosis in mice
Source: Nat Commun. 2026 Mar 3;17:3389. doi: 10.1038/s41467-026-70136-5 (PMC13065827; doi:10.1038/s41467-026-70136-5)
Supplement: Supplementary file 2 — Reporting summary [file 41467_2026_70136_MOESM2_ESM.pdf]

nature portfolio

Corresponding author(s): Haojie Yu

Last updated by author(s): Feb 2, 2026

Reporting Summary

Nature Portfolio wishes to improve the reproducibility of the work that we publish. This form provides structure for consistency and transparency in reporting. For further information on Nature Portfolio policies, see our Editorial Policies and the Editorial Policy Checklist.

Statistics

For all statistical analyses, confirm that the following items are present in the figure legend, table legend, main text, or Methods section.

n/a	Confirmed	
☐	☒	The exact sample size (*n*) for each experimental group/condition, given as a discrete number and unit of measurement
☐	☒	A statement on whether measurements were taken from distinct samples or whether the same sample was measured repeatedly
☐	☒	The statistical test(s) used AND whether they are one- or two-sided<br>*Only common tests should be described solely by name; describe more complex techniques in the Methods section.*
☐	☒	A description of all covariates tested
☐	☒	A description of any assumptions or corrections, such as tests of normality and adjustment for multiple comparisons
☐	☒	A full description of the statistical parameters including central tendency (e.g. means) or other basic estimates (e.g. regression coefficient) AND variation (e.g. standard deviation) or associated estimates of uncertainty (e.g. confidence intervals)
☐	☒	For null hypothesis testing, the test statistic (e.g. *F*, *t*, *r*) with confidence intervals, effect sizes, degrees of freedom and *P* value noted<br>*Give P values as exact values whenever suitable.*
☒	☐	For Bayesian analysis, information on the choice of priors and Markov chain Monte Carlo settings
☒	☐	For hierarchical and complex designs, identification of the appropriate level for tests and full reporting of outcomes
☒	☐	Estimates of effect sizes (e.g. Cohen's *d*, Pearson's *r*), indicating how they were calculated

Our web collection on statistics for biologists contains articles on many of the points above.

Software and code

Policy information about availability of computer code

Data collection	High-content fluorescence images were acquired on a PerkinElmer Operetta system running Harmony v4.9 (PerkinElmer)<br>For liver metabolomics, hydrophilic interaction liquid chromatography (HILIC) analyses of water-soluble metabolites in positive ion mode (HILIC-pos) were performed on a Shimadzu Nexera X2 U-HPLC (Shimadzu) coupled to a Q Exactive hybrid quadrupole Orbitrap mass spectrometer (Thermo Fisher Scientific). HILIC analyses in negative ion mode (HILIC-neg) were conducted on a Waters AQUITY UPLC system (Waters, Milford, MA) coupled to a 5500 QTRAP mass spectrometer (SCIEX, Framingham, MA). Positive ion mode analyses of polar and non-polar plasma lipids (C8-pos) were performed on a Shimadzu Nexera X2 U-HPLC coupled to an Exactive Plus Orbitrap mass spectrometer (Thermo Fisher Scientific, Waltham, MA). Reversed-phase chromatography in negative ion mode (C18-neg) for free fatty acids, bile acids, and intermediate-polarity metabolites was conducted using a Shimadzu Nexera X2 U-HPLC coupled to a Q Exactive Orbitrap mass spectrometer (Thermo Fisher Scientific). Raw data from Q Exactive/Exactive Plus instruments were processed with TraceFinder (Thermo Fisher Scientific) and Progenesis QI (Nonlinear Dynamics), while 5500 QTRAP data were processed with MultiQuant (SCIEX).<br>Liver and adipose tissue microarray data were collected using the Affymetrix GeneChip™ Mouse Gene 2.1 ST Array Strip according to the manufacturer's instructions.<br>Phosphoproteomics datasets were acquired by reverse-phase LC on an Easy-Spray column (50 cm × 75 µm, 2 µm; Thermo Fisher Scientific) using an nLC1000 UHPLC system coupled to an Orbitrap Fusion Lumos mass spectrometer (Thermo Fisher Scientific). A 70-min water/0.1% formic acid–acetonitrile/0.1% formic acid gradient was applied. Data-dependent acquisition was performed in positive-ion mode (MS1: Orbitrap 60,000 resolution at m/z 200; 350–1550 m/z; AGC target 4×10^5. MS2: Orbitrap HCD at 42% NCE; AGC target 7.5×10^4; 1 m/z isolation window; 100 ms maximum injection time).
Data analysis	Fluorescence images of BODIPY-stained adipocytes and brightfield images of H&E-stained adipose tissue were analyzed in ImageJ (NIH) using the AdipoSoft plugin to quantify adipocyte size.

For microarray data, the differential expression results (DEG tables) were provided by the core facility; we used the provided DEG lists for downstream interpretation and figure generation.
Metabolomics data were processed from raw files using TraceFinder (Thermo Fisher Scientific) and Progenesis QI (Nonlinear Dynamics) for datasets acquired on Q Exactive/Exactive Plus instruments, while data from the 5500 QTRAP system were analyzed using MultiQuant (SCIEX).
For phosphoproteomics analysis, processed differential expression tables were provided by the core facility; Downstream analysis used kinase–substrate enrichment analysis (KSEA) to predict upstream kinases and estimate relative kinase activity changes from phosphosite data.

For manuscripts utilizing custom algorithms or software that are central to the research but not yet described in published literature, software must be made available to editors and reviewers. We strongly encourage code deposition in a community repository (e.g. GitHub). See the Nature Portfolio guidelines for submitting code & software for further information.

Data

Policy information about availability of data

All manuscripts must include a data availability statement. This statement should provide the following information, where applicable:

- Accession codes, unique identifiers, or web links for publicly available datasets
- A description of any restrictions on data availability
- For clinical datasets or third party data, please ensure that the statement adheres to our policy

The microarray data generated in this study have been deposited in the Gene Expression Omnibus (GEO) database under accession number GSE316218.

Research involving human participants, their data, or biological material

Policy information about studies with human participants or human data. See also policy information about sex, gender (identity/presentation), and sexual orientation and race, ethnicity and racism.

Reporting on sex and gender	NA
Reporting on race, ethnicity, or other socially relevant groupings	NA
Population characteristics	NA
Recruitment	NA
Ethics oversight	This study did not involve the recruitment of human participants or the collection of human samples. The Simpson–Golabi–Behmel syndrome (SGBS) preadipocyte cell line used in this study is a previously established human cell line that was derived with informed consent and approved by the Ethical Committee of the University of Ulm, as described in the original publication. The cell line was kindly supplied by Martin Wabitsch (University of Ulm, Germany). No additional ethical approval was required for the present study.

Note that full information on the approval of the study protocol must also be provided in the manuscript.

Field-specific reporting

Please select the one below that is the best fit for your research. If you are not sure, read the appropriate sections before making your selection.

☒ Life sciences ☐ Behavioural & social sciences ☐ Ecological, evolutionary & environmental sciences

For a reference copy of the document with all sections, see nature.com/documents/nr-reporting-summary-flat.pdf

Life sciences study design

All studies must disclose on these points even when the disclosure is negative.

Sample size	Sample sizes were chosen based on previous studies in the field and standard practice in molecular and cellular biology and also animal experiments. No statistical method was used to predetermine sample size, but sample numbers were sufficient to detect biologically relevant differences and were consistent with prior publications.
Data exclusions	No data were excluded from the analyses.
Replication	All key experiments were independently replicated at least three times with consistent results. Data shown are representative of at least three independent experiments unless otherwise stated.
Randomization	Animals were assigned to experimental groups according to genetic status (knockout or knockdown) with wild-type controls.
Blinding	Outcome assessments, including histological quantification and image analysis, were performed by investigators blinded to group identity.

Blinding | Blinding was implemented to ensure objective evaluation and minimize assessment bias.

Reporting for specific materials, systems and methods

We require information from authors about some types of materials, experimental systems and methods used in many studies. Here, indicate whether each material, system or method listed is relevant to your study. If you are not sure if a list item applies to your research, read the appropriate section before selecting a response.

Materials & experimental systems

n/a	Involved in the study
☐	☒ Antibodies
☐	☒ Eukaryotic cell lines
☒	☐ Palaeontology and archaeology
☐	☒ Animals and other organisms
☒	☐ Clinical data
☒	☐ Dual use research of concern
☒	☐ Plants

Methods

n/a	Involved in the study
☒	☐ ChIP-seq
☒	☐ Flow cytometry
☒	☐ MRI-based neuroimaging

Antibodies

Antibodies used

-Mouse monoclonal anti-HSP90 (Santa Cruz Biotechnology, sc-13119)
-Rabbit polyclonal anti- Akt (Cell Signaling Technology, #9272)
-Rabbit monoclonal anti-phospho-Akt(Ser473)(D9E) (Cell Signaling Technology, #9271)
-Rabbit monoclonal anti-PPARγ (C26H12) (Cell Signaling Technology, #2435)
-Rabbit polyclonal anti-C/EBPα [EP708Y] (Abcam, ab40761)
-Mouse monoclonal anti-C/EBPα(D5) (Santa Cruz Biotechnology, sc-365318)
-Mouse monoclonal anti-β-actin (Sigma-Aldrich, A5316)
-Rabbit monoclonal anti-p44/42 MAPK (Erk1/2) (Cell Signaling Technology, #4695)
-Rabbit monoclonal anti-phospho-p44/42 MAPK (Erk1/2) (Thr202/Tyr204) (Cell Signaling Technology, #4370)

Validation

All primary antibodies used in this study were validated by the manufacturers for the relevant species and applications, as indicated on their product datasheets.No additional validation was performed for secondary antibodies.

Eukaryotic cell lines

Policy information about cell lines and Sex and Gender in Research

Cell line source(s)

The Human Simpson–Golabi–Behmel syndrome (SGBS) cell line was kindly supplied by Martin Wabitsch (University of Ulm, Germany).

Authentication

SGBS cells were authenticated as preadipocytes based on their response to adipogenic cues (cocktails) and the expression of adipogenic marker genes.

Mycoplasma contamination

All cell lines and primary cells tested negative for mycoplasma contamination using PCR-based methods.

Commonly misidentified lines (See ICLAC register)

None of the cell lines used are listed in the ICLAC register of commonly misidentified cell lines.

Animals and other research organisms

Policy information about studies involving animals; ARRIVE guidelines recommended for reporting animal research, and Sex and Gender in Research

Laboratory animals

Whole-body Gpr146 knockout (Gpr146-/-), adipose-specific knockout (Adipoq-Cre+ Gpr146fl/fl) and liver-specific knockout (Alb-Cre+ Gpr146fl/fl) mice, together with their corresponding control littermates including Gpr146+/+, Adipoq-Cre- Gpr146fl/fl and Alb-Cre- Gpr146fl/fl mice were generated as described in our previous study (DOI: 10.1016/j.cell.2019.10.034). C57BL/6J (RRID: IMSR_JAX:000664) and B6J.129(Cg)-Gt(ROSA)26Sortm1.1(CAG-cas9*,-EGFP)Fezh/J (RRID: IMSR_JAX:026179) were obtained from Jackson Laboratory.

Wild animals

This study did not involve wild animals.

Reporting on sex

Both sexes of the knockout mouse models were used in this study. For the acute depletion of Gpr146, only male mice were employed.

Field-collected samples

This study did not involve samples collected from the field.

Ethics oversight	All animal care and experimental procedures conducted in this study received approval from the Institutional Animal Care and Use Committee of Harvard University and National University of Singapore.

Note that full information on the approval of the study protocol must also be provided in the manuscript.

Plants

Seed stocks	NA
Novel plant genotypes	NA
Authentication	NA
